# Synchronization and Propagation of Global Sleep Spindles

**DOI:** 10.1371/journal.pone.0151369

**Published:** 2016-03-10

**Authors:** Rafael Toledo Fernandes de Souza, Günther Johannes Lewczuk Gerhardt, Suzana Veiga Schönwald, José Luiz Rybarczyk-Filho, Ney Lemke

**Affiliations:** 1 Departamento de Física e Biofísica, UNESP - Univ. Estadual Paulista, Botucatu, Brazil; 2 Departamento de Física e Química da Universidade de Caxias do Sul, Caxias do Sul, Brazil; 3 Hospital de Clínicas de Porto Alegre (HCPA), Neurology and Pulmonology Sections, Porto Alegre, Brazil; University of Salamanca- Institute for Neuroscience of Castille and Leon and Medical School, SPAIN

## Abstract

Sleep spindles occur thousands of times during normal sleep and can be easily detected by visual inspection of EEG signals. These characteristics make spindles one of the most studied EEG structures in mammalian sleep. In this work we considered global spindles, which are spindles that are observed simultaneously in all EEG channels. We propose a methodology that investigates both the signal envelope and phase/frequency of each global spindle. By analysing the global spindle phase we showed that 90% of spindles synchronize with an average latency time of 0.1 s. We also measured the frequency modulation (chirp) of global spindles and found that global spindle chirp and synchronization are not correlated. By investigating the signal envelopes and implementing a homogeneous and isotropic propagation model, we could estimate both the signal origin and velocity in global spindles. Our results indicate that this simple and non-invasive approach could determine with reasonable precision the spindle origin, and allowed us to estimate a signal speed of 0.12 m/s. Finally, we consider whether synchronization might be useful as a non-invasive diagnostic tool.

## 1 Introduction

Synchronization is a robust and widespread phenomenon in complex systems that usually occurs in oscillatory interacting units. There are many examples of synchronization in various cells and organisms, including cardiac pacemaker cells, rhythmically flashing fireflies, chorusing crickets and neurons [1]. Since Crick and Koch proposed that conscience phenomena could be related to synchronization of different cerebral regions that impose a temporary global unity to the brain [2], several other studies have investigated the role that synchronization plays in different aspects of neuronal physiology [3–9].

Electroencephalography (EEG) measures electrical oscillations of the brain through electrodes placed on the scalp [10]. This technique can be seen as a non-invasive window into the internal rhythms of the brain. EEG signals are rich in detail, and by nature are non-stationary [11–13]. Information gained from EEG during sleep may be even richer than measurements taken during wakefulness, and EEG represents an important method to assess normal and altered physiology because sleep EEG transients may be affected by some diseases. Of all EEG elements, sleep spindles (SS) are certainly the most studied short transient [14, 15]. SS were first documented in the 1930s [16] and are now known to be associated with memory processing and learning [17–19].

SS are defined as wave packets between 11 Hz and 16 Hz that have a duration of less than 2 s and primarily occur during non-REM (NREM) sleep stage II, although they may also occur during other sleep stages [20, 21]. SS can be represented as an oscillatory function (wavefunction) with an average frequency modulated by an envelope [14]. Although this envelope is not exactly Gaussian for true SS, this model can nevertheless be used to describe the time limits for their occurrence [14, 22]. By determining the ratio between this wave and its envelope, we can perform a normalization to retain only the oscillatory component of the signal. As such, the signal envelope can be used for wave propagation analysis (spread across electrodes) and the oscillatory component can be applied to synchrony analysis. SS can be easily detected and characterized, which allows an independent investigation of envelopes and oscillatory behaviors. Thus, SS are an excellent model for evaluating brain synchronization. The widespread availability and non-invasiveness of EEG could allow it to be used to explore the relevance of synchronization issues to different mental diseases, provided that powerful and automated accompanying diagnostic tools can be developed.

During the course of one night’s sleep, spindles appear thousands of times and they likely have a thalamic origin. Moreover, scalp-based measurement of SS can reflect the activity of the reticulo-thalamic-cortex communication network [23–25]. SS have rich behaviors with amplitude and frequency modulation (short time chirp) that shows high non-stationarity [22, 26, 27]. Indeed, there are two types of SS, slow and fast, which appear to have different physiological characteristics [14]. In a study using Ca and Na channel-blocking drugs, different pharmacological responses for fast and slow spindles have suggested that different neuronal populations may be implicated in the production of those two spindle types [28]. It is worthwhile to note that a systematic increase in negative SS chirp factor can be associated with a shift towards lower frequencies if the FFT power spectrum is analyzed. As with any event detected on the surface of the scalp, SS represent the oscillations of a large set of neurons, and are prone to spread from a central point of occurrence to other locations across the brain surface [29–33]. Depth recordings have shown that low voltage spindles may occur in different cortical areas in a local, asynchronous manner, whereas higher voltage spindles may behave as diffuse phenomena [34]. As such, SS are complex events that may be simultaneously measured at different scalp positions and, at the same time, behave like a propagating wave. In recent years SS propagation and frequency modulation (chirp) have been studied in greater detail [18, 19, 22, 27]. Given the diffuse nature of SS, it is relevant to determine whether the different brain regions that participate in global spindles present some level of phase synchronization and how this synchronization relates to dynamic variables associated with SS.

The aim of this work was to extract unambiguous, high voltage, diffuse scalp EEG spindles from human subjects and to investigate spindle dynamics in terms of phase synchronization, chirp and signal propagation, while taking into consideration slow and fast spindle types. A set of independent methodologies showing reciprocally consistent results was used in this work. We believe that this set of procedures (rather than only one methodology) can be useful because of the lack of stationarity inherent to SS segments. We will measure both envelope and oscillatory characteristics to investigate how SS propagate through the brain and discuss how these characteristics are correlated.

## 2 Methods

### 2.1 Subjects and Sleep Studies

The eight subjects included in the present study were selected from a database that was previously designed for studies on spindle characteristics in obstructive sleep apnea (OSA) [27, 35]. Consecutive patients aged 34–60 with clinically suspected OSA [20] were prospectively enrolled for polysomnography (PSG) at a university hospital-based sleep clinic between April 2007 and July 2009 (for additional details see [35]). Subjects provided informed written consent and the study was approved by the local ethics committee (Comitê de Ética do Grupo de Pesquisa e Pós-Graduação do Hospital das Clínicas de Porto Alegre, GPPG HPCA approval number: 100248). Six of these patients were considered to be non-OSA subjects (global Apnea-Hypopnea Index, AHI below 5). Subject 7 was a 54 year-old woman with body mass index 40 and mild lung emphysema. Subject 8 was a 43 year-old male with moderate OSA (AIH 15.9). Patients were taking fluoxetine (1), amitriptyline (1) and several non-psychotropic drugs for non-neurological co-morbidities.

Continuous recordings were performed during the usual sleep period (23:00-07:00 h) on a 16 bit resolution digital system (Deltamed, Racia-Alvar, France). The recording protocol followed standard guidelines [21] and included information on scalp EEG, eye movement, chin and leg electromyograms, electrocardiograms, snoring, airflow by oronasal thermistor, thoracic and abdominal respiratory effort, body position and pulse oximetry. Silver electrodes were placed over 10 standard 10–20 IS EEG positions (F3, C3, P3, O1, A1, F4, C4, P4, O2, A2). Initial impedances were below 10 K*Ω*. The signal was acquired with a 256Hz sampling rate, filtered at 0.5–35Hz and analyzed off-line using Coherence 3NT software version 4.4 (Deltamed, France). Electrodes were referenced to contralateral auricular positions according to AASM 2007 recommendations, with ground electrode placed on the forehead. Signal analysis was performed on left and right frontal (F3, F4), central (C3, C4) and parietal (P3, P4) EEG channels referenced to (A1+A2)/2. Sleep stages, arousals and respiratory events were visually scored by a trained polysomnographer in accordance with standard recommendations, and applying obstructive hypopnea rule 4B [21].

### 2.2 Sleep Spindle Detection and Selection

Spindle detection was carried out with a matching pursuit (MP) program obtained from http://eeg.pl [36]. MP was used exclusively to identify SS candidates and was applied only in NREM sleep stages II and III. All subsequent analyses were performed on the original time series (without subsampling). MP has been previously described in detail [37, 38] and was suitable for sleep spindle representation [22, 36, 39–41]. After subsampling to 128Hz, each whole-night EEG series was segmented into juxtaposed bins of 2048 digital points and subjected to MP decomposition with a dictionary size of 10^5^ atoms, stopping at 96 iterations. Each atom obtained with MP has a central point in time and frequency, and time and frequency full widths at half maximum (FWHM) corresponding to ±*σ* on a gaussian curve. FWHM duration was used as one parameter for atom selection. Atoms with FWHM duration between 0.5s and 2s and central frequency between 11Hz and 16Hz, hereafter called spindles, were collected in the procedure. Notably, individual MP atom fulfilling detection criteria is not conceptually equivalent to a visual sleep spindle, and the procedure is robust and reliable at a statistical level [41]. The number of atoms that obey these criteria is usually on the average of 2,000/channel/night. Each extracted segment had a 2 s duration, with the highest amplitude SS positioned on the center.

To ensure inclusion of high voltage diffuse spindles while avoiding inclusion of overlapping spindles, only the 100 highest-amplitude, isolated MP atoms were selected for each subject, regardless of electrode position. The electrode containing this atom was defined as the leading electrode.

Segments with artifacts on any channel were discarded. Some EEG segments that still contained multiple superimposed frequency peaks on any given channel were *a posteriori* discarded (see below in Synchrony Across Channels subsection). In that case, the next highest amplitude atom was chosen in order to reach 100 SS for each subject. Therefore, the total segment number obtained was 760 × 8 channels (subject number 2 contributed only 60 SS due to noise).

### 2.3 Signal Envelope

**Fig 1:**
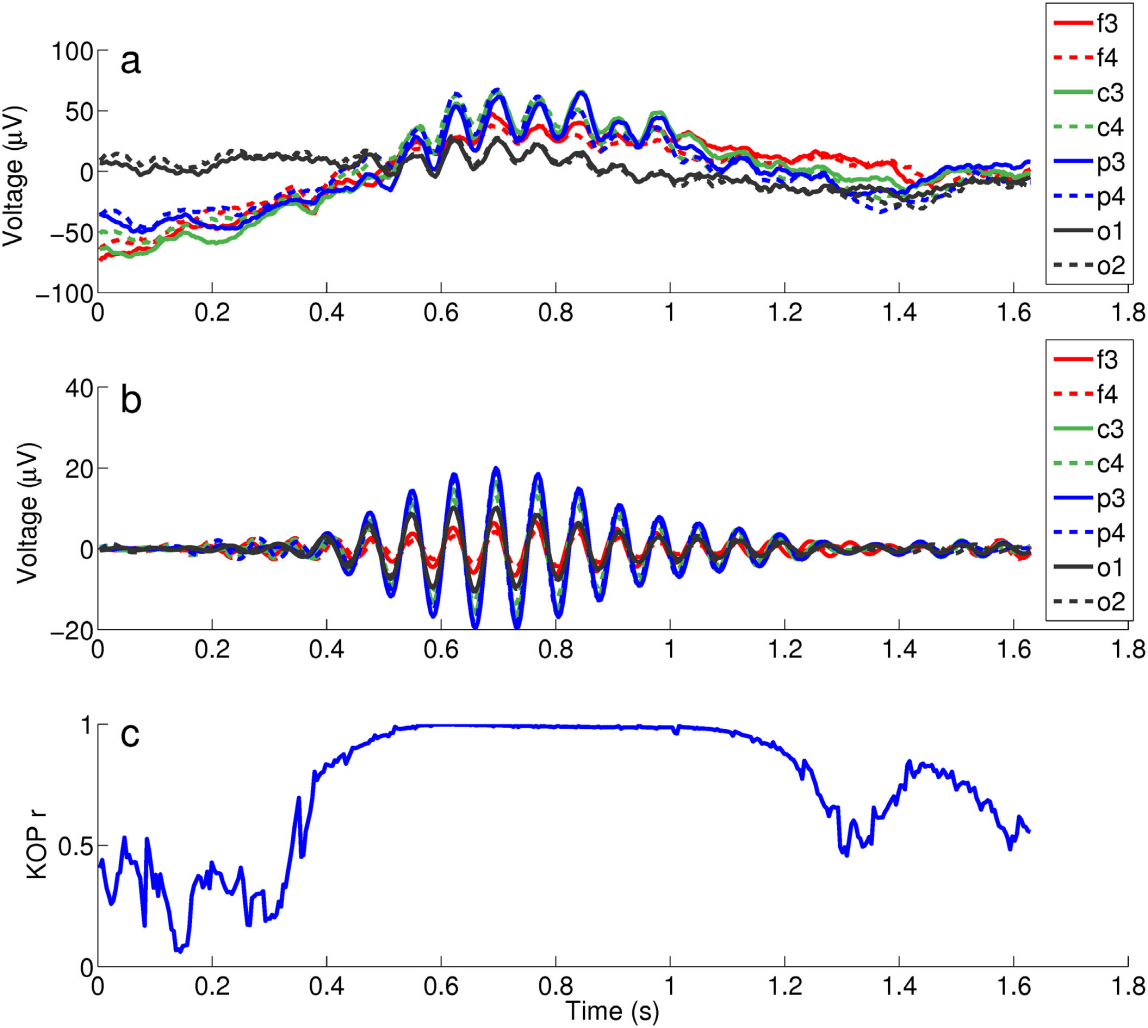
Methodology applied to determine KOP. (a) 2 s segment signal with the SS located in the middle; (b) raw signal filtered between 11–16 Hz; (c) KOP as a function of time calculated for that set of simultaneous signals. The synchronization starts in about 0.4 s and ends in 1.2s.

Initially we used EEG signals corresponding to SS segments,$S_{orig}^{k}$, from each evaluated channel *k* (F3, F4, C3, C4, P3, P4, O1, O2) and submittted them to a filter as shown in Fig 1a and 1b. Signals from each channel were then normalized using Eq 1:

$$S^{k}\left(t\right)=F\left\{S_{orig}^{k}\left(t\right)\right\}-\langle S^{k}\left(t\right)\rangle$$ (1)

where the average of SS intervals was taken. The envelope for *S*^*k*^ was obtained by numerically estimating |*S*^*k*^(*t*)| local maxima and generating an interpolated curve, *M*^*k*^(*t*). The envelope center was defined as max(*M*^*k*^) and SS duration was defined as the envelope Full Width Half Maximum (FWHM).

We also defined a phase, *θ*_*j*_(*t*), to characterize the oscillatory part of the signal (Eq 2):

$$\cos\theta_{j}\left(t\right)=\frac{S^{j}\left(t\right)}{M^{j}\left(t\right)}$$ (2)

### 2.4 Synchrony Across Channels

Detection of SS phase synchrony across different EEG channels was measured using an adaptation of standard methodologies [8]. Our methodology was based on the determination of the Kuramoto Order Parameter (KOP) [42, 43], which in this study was calculated using the Eq 3:

$$r\left(t\right)=\left|\frac{1}{N}\sum_{k=1}^{N}e^{i\theta_{k}\left(t\right)}\right|,$$ (3)

where *θ*_*j*_ are signal phases for each (*N* = 8) of the channels.

To dynamically characterize the synchronization process we fitted *r*(*t*) by Eq 4:

$$r\left(t\right)=A [\arctan\left(\frac{t-t_{0}}{T}\right)-\arctan\left(\frac{t-t_{0}-m}{T}\right)],$$ (4)

where A is the amplitude of the KOP parameter on the window interval; *t*_0_ is the time shift for a given SS; *T* is the synchronization time; and *m* is synchronization duration. This functional form was chosen for its convenience.

KOP evolution can be illustrated for a SS detected on different channels (Fig 1). In the specific case shown in (Fig 1a–1c), there is an increase in synchrony near 0.5 s that ends approximately one second later, around 1.5 s.

When the *r*(*t*) parameter presented as an irregular shape, the corresponding SS was discarded and the subsequent element was chosen. Residual multiple frequency peak SS (possibly representing superimposed SS [14]) were thereby removed from the sample. This procedure ensured inclusion of elements that had high interchannel synchronization duration (high *m*), which are expected to correspond to diffuse spindles.

### 2.5 Spindle Frequency Analysis

Fast Fourier Transform (FFT) was used in order to determine an average frequency for each 512-point SS segment (0.5 Hz resolution). SS frequency was defined for each channel as the frequency corresponding to the highest power spectrum peak in the 11–16 Hz range. Global SS frequency was the frequency mean for all eight channels. Diffuse spindles were divided into two groups according to average frequency, Slow (*f* ≤ 13 Hz) and Fast (*f* > 13 Hz).

In order to further verify whether the selected SS sample predominantly comprised diffuse or local spindles, a correlation analysis of SS frequency was performed between each of two channels, and considering the original SS segments corresponding to each channel. This procedure was then repeated for randomly chosen segments from each of the two channels. There was high interchannel frequency correlation for original SS segments, which was lost when random segments were analyzed.

### 2.6 Chirp Measurement

Frequency modulation (also called chirp) was calculated by windowed FFT (WFT) applied to the filtered signal. For each signal segment, the 512-point signal was extended by 0.5 s both before and after the original signal. In this 3 s segment, a 2 s moving window was used to multiply the series by a Gaussian function centered on the middle of the window and divided into 13 steps. This Gaussian procedure was chosen in order to best estimate SS central frequency along the time frame. FFT was evaluated for each 2 s segment and the frequency at spectrum peak was analyzed to yield 13 frequency peaks along the time frame. These 13 frequency peaks were used to fit a linear slope that was considered as the spindle chirp value (0.25 Hz/s resolution).

The chirp value distribution for slow and fast spindles was analyzed for each channel (single channel chirp distribution), and the average chirp value for each SS across all channels (channel-averaged chirp distribution) was also determined. A Student’s t test was performed to assess the null hypothesis that chirp value distribution had a zero mean.

### 2.7 Signal Propagation

We assumed a very simple model for spindle propagation. We considered that each SS is produced at a single point at position $\vec{r_{k}}$ and propagates as a spherical wave with velocity *v*_*k*_. Thus, using the electrode positions and their relative distances, we compared the time delay response (*τ*) obtained from these electrodes to find the most probable origin and propagation velocity of a given SS.

To investigate the delay *τ*, initially we considered the cross-correlation function (Eq 5):

$$h\left(t\right)=\int_{t_{o}}^{t_{f}}M^{i}\left(t^{\prime }\right)M^{j}\left(t^{\prime }-t\right)dt^{\prime },$$ (5)

*τ* was then defined by Eq 6

max(h(t))=h(τ) (6)

The delay *τ* represents the time delay with maximal overlap between both signals. The spherical model allows us to infer time delays for any two channels *i* and *j* (Eq 7):

$$\vartheta_{ij}^{k}=\frac{|\vec{r_{k}}-\vec{R_{i}}\left|-\right|\vec{r_{k}}-\vec{R_{j}}|}{v_{k}}$$ (7)

where *R* represents channel positions. These quantities could be directly compared to delays measured experimentally for each SS *k*, $\tau_{i,j}^{k}$.

For each physiologically viable $\vec{r}$ and *v* we defined:

$$\epsilon_{k}\left(\vec{r},v\right)=\sum_{ij}{\left(\vartheta_{ij}^{k}-\tau_{ij}^{k}\right)}^{2}$$ (8)

By minimizing *ϵ* with respect to position and velocity, we can infer position $\vec{r_{k}^{*}}$ and velocity $v_{k}^{*}$

$$\min_{\vec{r},v}\epsilon_{k}\left(\vec{r},v\right)=\epsilon_{k}\left(\vec{r^{*}},v^{*}\right)$$ (9)

Channel positions and relative distances were estimated by positioning electrodes in a generic MRI regular head examination (obtained from the BIRN database [44]) using the 10–20 IS standard [45]. As no study subject had an abnormal head size or cranial asymmetry, the distances between the electrodes were assumed to be constant among the different subjects and the error measurement was estimated to be 0.01 m.

**Fig 2:**
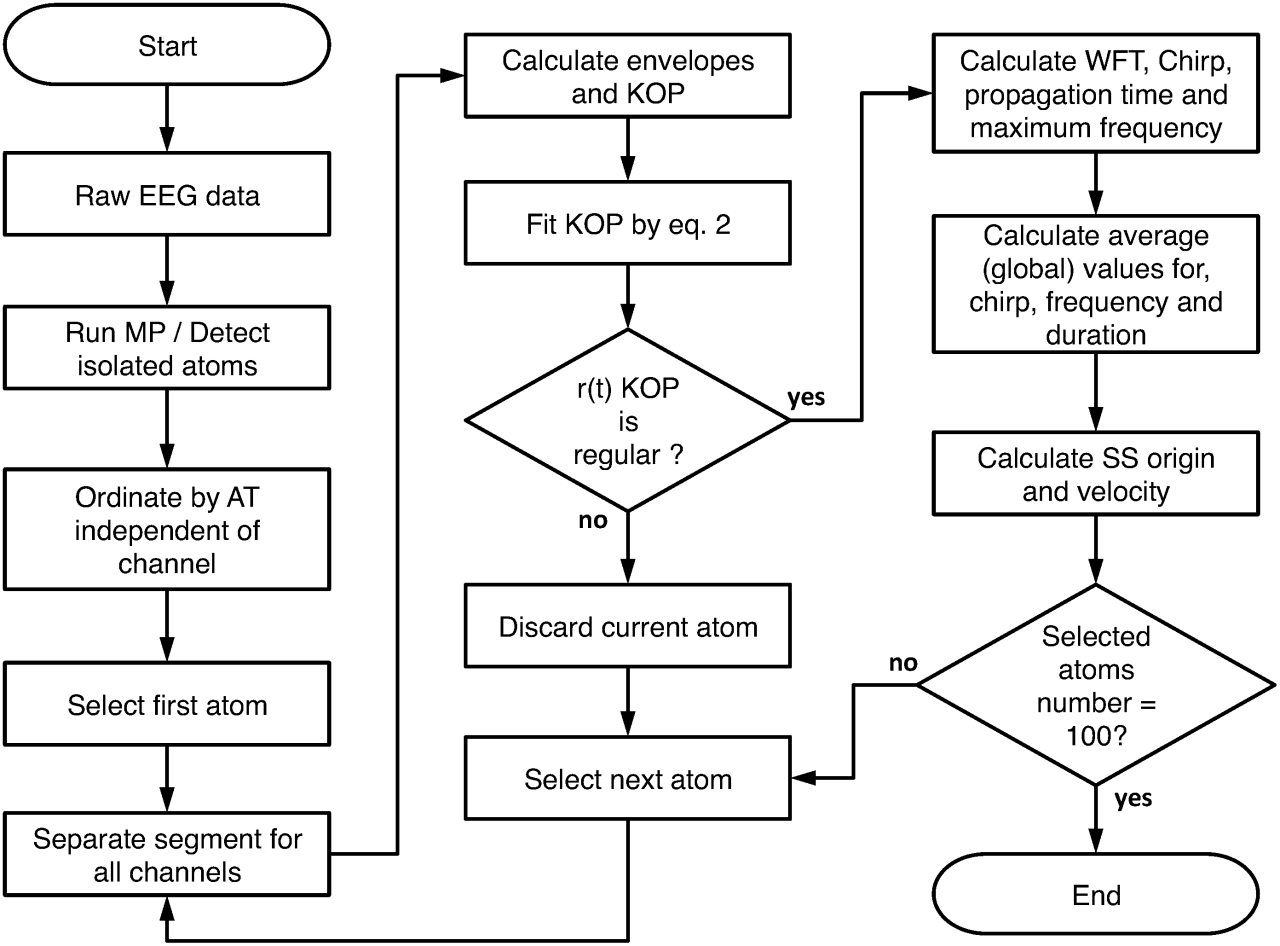
Flowchart of SS parameter analysis.

A detailed workflow of the proposed methodology for measuring all of the relevant parameters is shown in Fig 2.

## 3 Results

### 3.1 Spindle frequency, duration, amplitude and chirp characteristics

**Fig 3:**
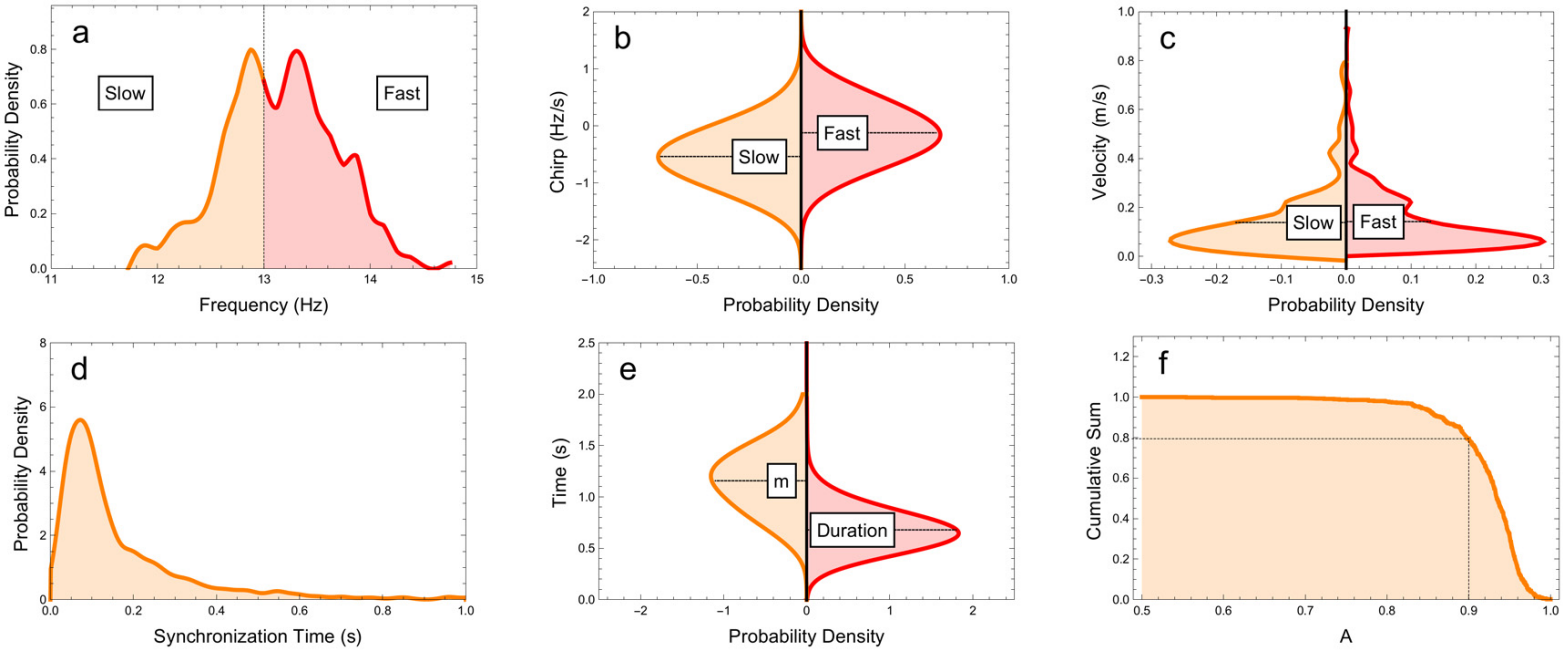
Histograms of measured parameters. Distribution of (a) frequencies, (b) chirp, (c) velocity, (d) synchronization time, (e) *m* (synchronization duration) and (sleep spindle) duration and (f) cumulative sum of *A* (synchronization parameter). We can observe that the frequency distribution is a bimodal function. The synchronization time average is larger than spindle duration, this behaviour indicates that the synchronization remains significant even for small signal amplitude. The slow spindles chirp is biased towards negative values while fast spindles distribution are evenly distributed around zero. Finally the amplitudes synchronization are concentrated on values close to 1, meaning that in general global spindles show significant phase locking behaviour. We could not detect any velocity differences for slow and fast spindles.

General descriptive parameters were obtained for 760 spindles (Fig 3 and Table 1).

**Table 1:** Mean values for spindle characterization metrics and KOP synchronization parameters. *T* represents the synchronization time, A amplitude, and *m* synchronization duration.

	Normality	Mean	Median	Std	InterQ
T	0.00	0.26	0.11	0.42	0.09
A	0.00	0.96	0.98	0.04	0.02
m	0.24	1.16	1.17	0.28	0.21
Duration	0.00	0.68	0.64	0.15	0.10
Velocity	0.00	0.14	0.12	0.14	0.08
Frequency	0.02	13.30	13.31	0.5	0.34
Slow Chirp	0.96	-0.61	-0.61	0.49	0.35
Fast Chirp	0.88	-0.17	-0.18	0.49	0.33

The frequency distribution (Fig 3a) was bimodal, with a valley between the peaks that was close to the predicted 13 Hz threshold that divided the slow and fast SS groups. On the leading channel, the median frequency of the SS was 13.3 Hz and the interquartile frequency range (IQ) was 0.3 Hz. The median duration of the SS was 0.64 s (0.10 s IQ).

The chirp distribution for slow and fast spindles in averaged channels was also determined (-0.61 Hz/s ±0.49 and - 0.17Hz/s ±0.49, respectively) and showed that slow spindles tended to have more negative chirp relative to fast spindles (Fig 3b). When single channel mean chirp values were analyzed, a distinctive antero-posterior gradient could be seen for both groups, with tendency of negative values and the highest p values (indicating the lowest probability of zero mean distribution) appearing in the electrodes that had a more anterior position (Fig 3b).

**Fig 4:**
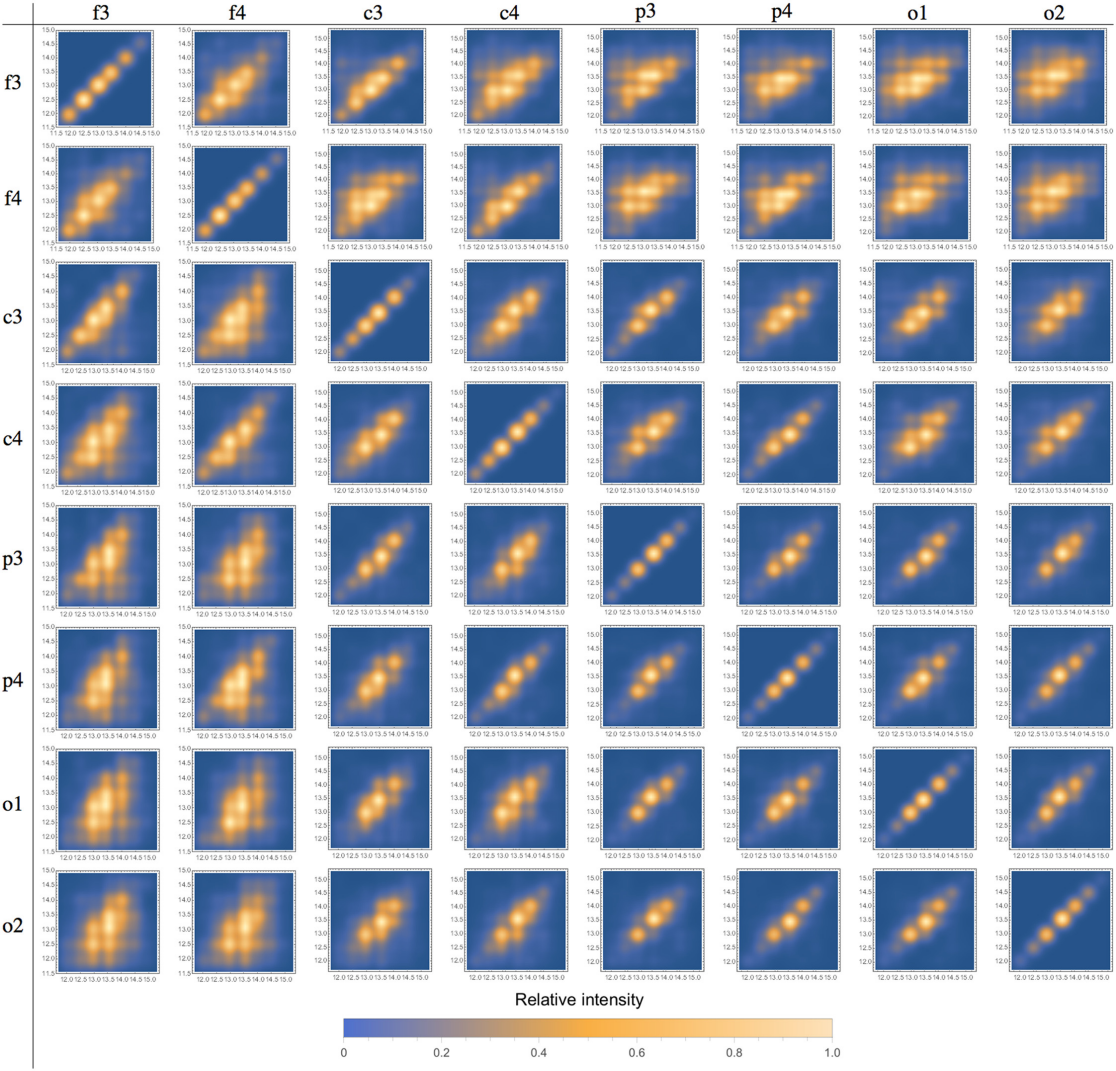
Scatter-plot for spindle frequencies in different channels. We can observe that correlation decreases as we consider more distant channels. Relative intensity is represented by the color scale bar displayed below.

**Fig 5:**
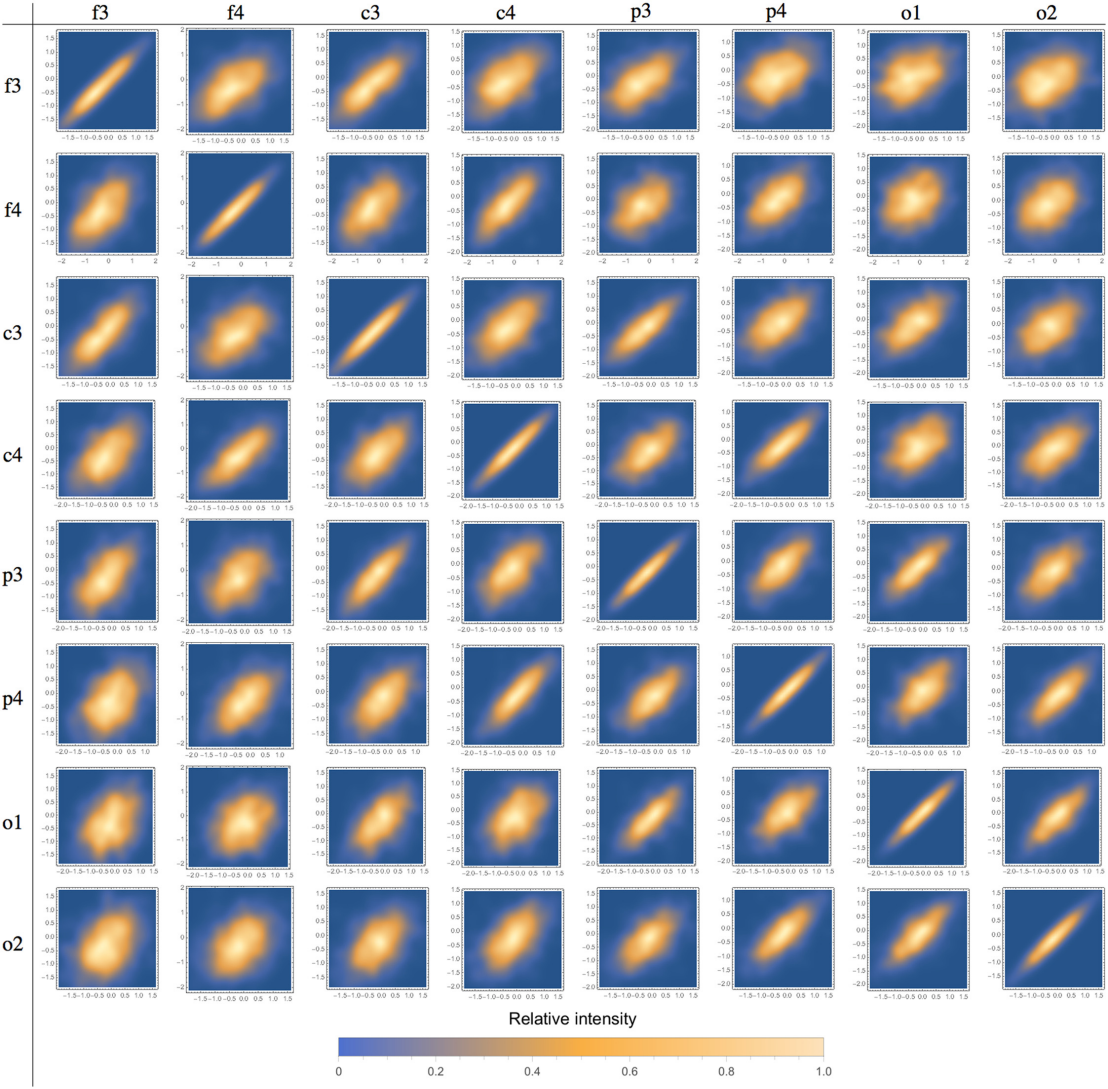
Scatter-plot for spindle chirp in different channels. We can observe that correlation decreases as we consider more distant channels. Relative intensity is represented by the color scale bar displayed below.

**Fig 6:**
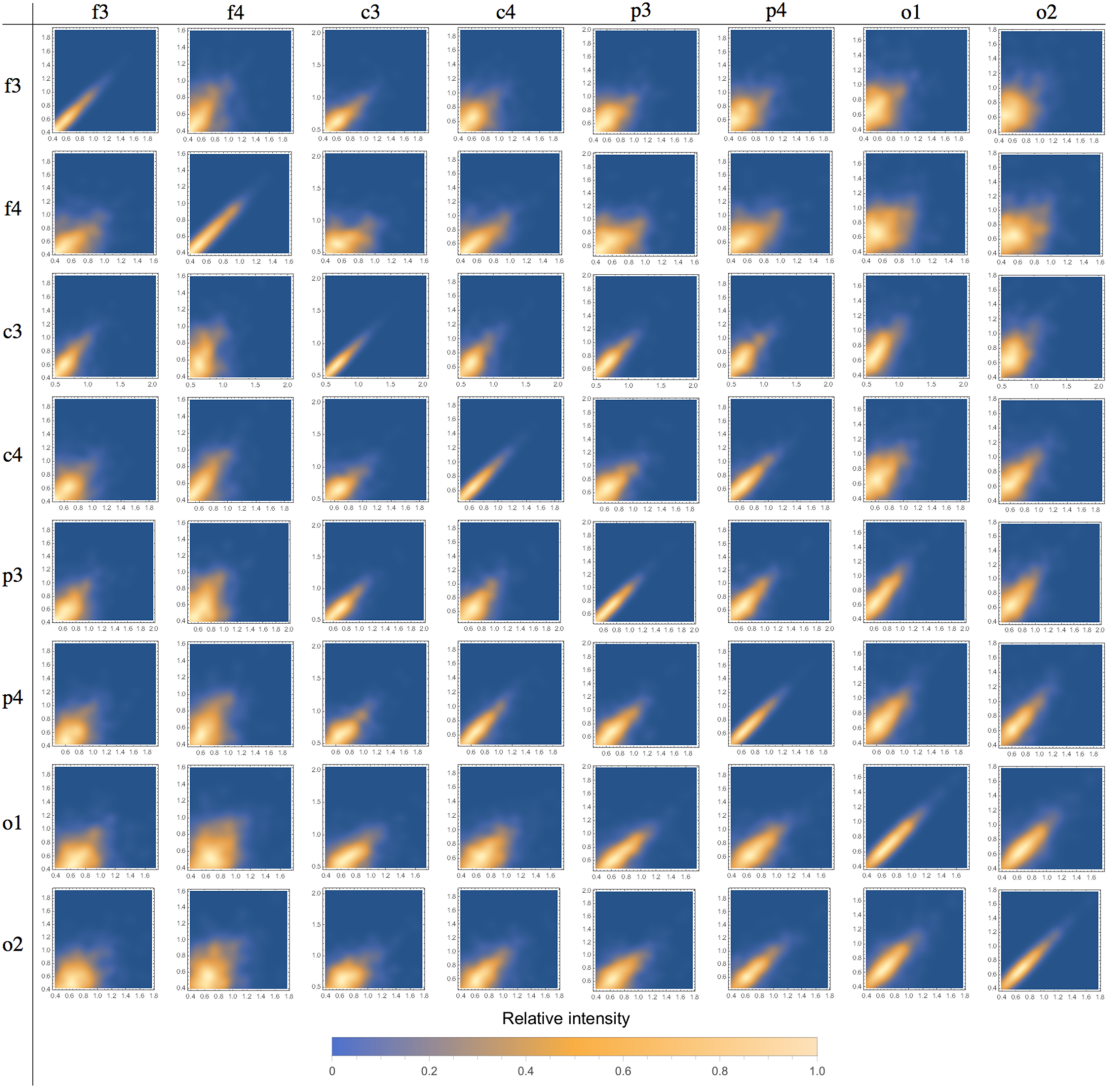
Scatter-plot for spindle durations in different channels. We can observe that correlation decreases as we consider more distant channels. Relative intensity is represented by the color scale bar displayed below.

We also evaluated the correlations among the measured parameters (Table 2). The most important correlations were chirp × *m*, chirp × frequency and duration × *m*. We found no relevant correlation between chirp and *A*, the synchronization intensity. Figs 4–6 show correlations of frequencies, chirp and durations among different channels.

**Table 2:** Correlation for metrics used in this paper. *T* represents synchronization time, A amplitude, and *m* synchronization duration. Correlations with p-value < 0.05 are in boldface. The most important correlations are chirp × *m*, chirp × frequency and duration × *m*.

	Frequency	Chirp	Velocity	T	A	m	Duration
Frequency	**1.00**	**0.44**	0.07	**-0.06**	0.08	0.04	-0.03
Chirp	**0.44**	**1.00**	0.01	0.07	0.00	**0.11**	**0.14**
Velocity	0.07	0.01	**1.00**	-0.05	**0.11**	-0.02	**-0.21**
T	**-0.06**	0.07	-0.05	**1.00**	**0.17**	0.04	**0.33**
A	0.08	0.00	**0.11**	**0.17**	**1.00**	**0.14**	**0.16**
m	0.04	**0.11**	-0.02	0.04	**0.14**	**1.00**	**0.49**
Duration	-0.03	**0.14**	**-0.21**	**0.33**	**0.16**	**0.49**	**1.00**

Inter-channel scatter-plots are displayed in Figs 4, 5 and 6 for frequency, chirp and velocity variables, respectively. The array in these figures forms two triangular matrices, symmetric in relation to the main diagonal (which represents the scatter-plot for the same channel, included just for comparison purposes). We can observe that correlations decrease as we compare more distant channels. The behavior is basically the same for all the considered variables. These results show that these spindles share most of its defining characteristics, they do not only occur at the same time, but are different manifestations of a single phenomenon. This is an indication that global and local spindles have distinct physiology, since most local spindles are not synchronous and are restricted to specific brain areas. The decreasing correlation indicates that the local population of neurons plays a role by making small but measurable perturbations on the detected signal. We do not observed any pattern on these variations, indicating that they might be intrinsically random [46].

### 3.2 Signal propagation and locating spindle generators

**Fig 7:**
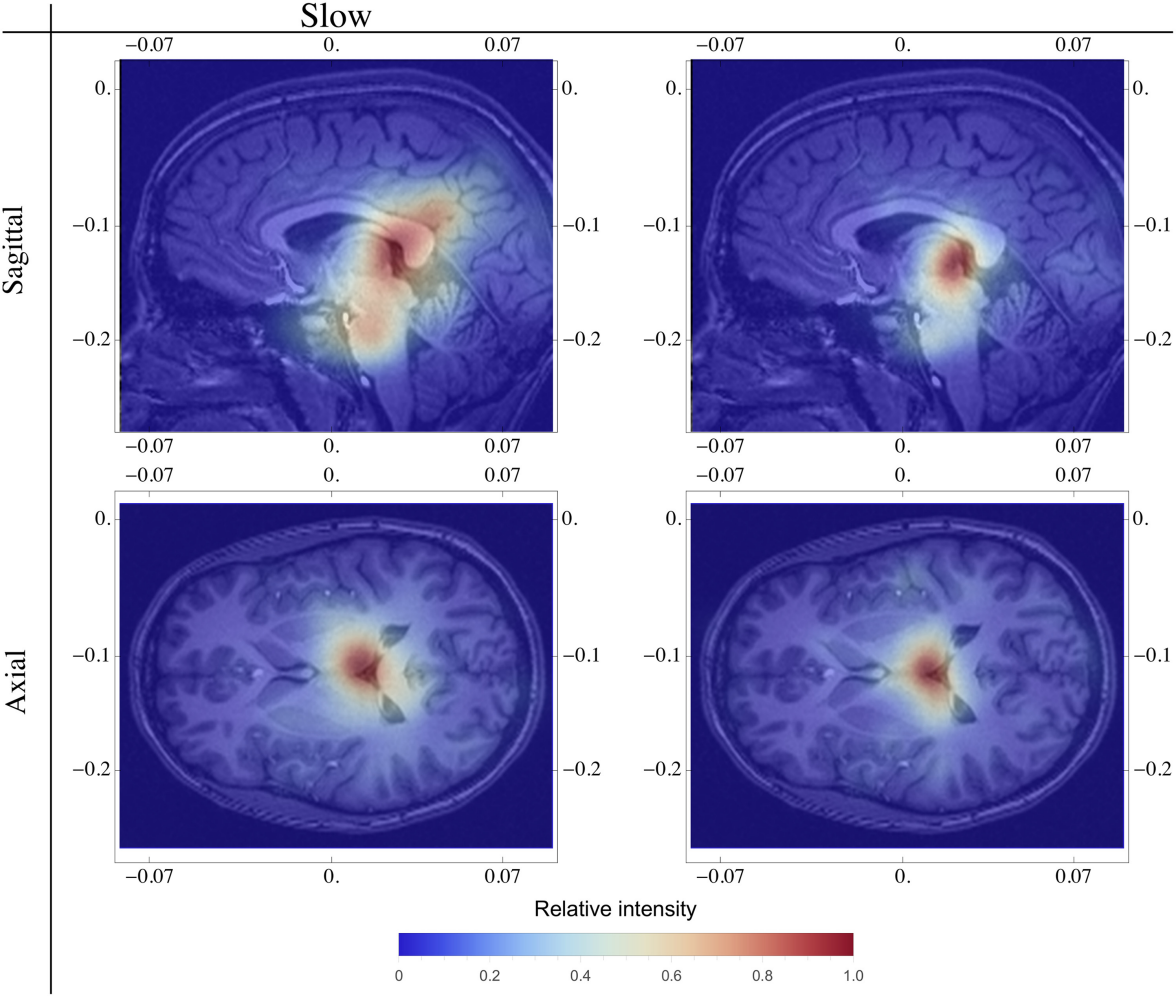
Heatmap for the origin of slow and fast spindles in axial and sagittal views. MRI images were obtained from the BIRN database [44]. Relative intensity is represented by the color scale bar displayed below. We can observe that in general the spindle origin is thalamic and, on the sagittal view, that slow SS origins have a broader distribution.

Upon calculating the signal propagation velocity distribution we found that the median propagation velocity was 0.12 m/s (0.08 m/s IQ, Fig 3c). By superimposing heatmaps atop MRI tomographic images deposited in the BIRN database, the SS propagation center location can be visualized in axial and sagital views (Fig 7). In these images the SS originates in the thalamic area, which is consistent with previous studies [23, 25, 47]. Thus, the proposed methodology generates results that are compatible with those obtained using much more sophisticated techniques [48]. However, we found no statistically significant difference in the origins of slow and fast SS spindles (data not shown).

### 3.3 Spindle synchrony across time

We next determined the distributions for spindle synchrony time (*T*) and synchrony duration (*m*) (Fig 3d and 3e, respectively). The SS duration as measured by FWHM was also evaluated (Fig 3e), as were the cumulative sums for synchrony amplitude (Fig 3f). The median synchronization time was 0.11 s (0.09 s IQ) and the spindle synchrony was fast and robust over time. The majority of spindles synchronized in less than 0.2 s, which is during the first oscillation. The median synchrony duration was 1.17 s (0.21 s IQ) and the median synchrony amplitude was 0.98 (0.02 IQ). Among all spindles, 80% had synchronization amplitudes that were larger than 90% (Fig 2f).

## 4 Discussion

EEG quantification can be challenging because of several factors, including a lack of stationarity, which also makes EEG useful for gathering electrophysiological information. As EEG analysis becomes more accurate and specific, signal tools are being created and their application for EEG is being carefully explored. Since small changes in EEG signals are known to reflect changes in internal brain mechanisms, such analysis tools take on added relevance from a diagnostic perspective. For example, a recent study showed that mice genetically predisposed to epilepsy display a gradient in the frequency modulation (chirp) that uniquely correlates with the development of the epileptic pathology [26]. The source of these signals may lie in how certain neural centers initialize and maintain their oscillation pattern.

In the present study, we used WFT to calculate SS chirp. In this approach the chirp measurement is independent from the MP spindle detection procedure and as such differs from earlier methodologies [22], wherein chirp measurement and spindle detection were performed using a modified MP dictionary that included Gabor chirplet functions. Nevertheless, our present results (slow chirps between 0.61 Hz/s ±0.49 and fast chirps between 0.17 Hz/s ±0.49) are in line with our earlier findings and reinforce the chirp effect [22, 27]. Slow SS had the clear tendency to chirp around 0.5 Hz/s, which is consistent with results from our previous study [22], also employing healthy subjects, but studying only a single (central) EEG channel.

In terms of synchronization data, we found that diffuse SS synchronize in 0.11 s, and this process seems to begin before the SS can be visually detected on the scalp. Our results indicate that synchronization could be starting earlier, and thus would have an effective duration that is longer than the measured SS duration. When macroscopic SS are captured on a scalp EEG, synchronization between channels appears to be already in the process of consolidation.

Although synchronization of signals in human brains has been measured previously, to the best of our knowledge this result is the first demonstration that phase synchronization can be measured for a SS. Such behavior should be a direct consequence of the large-scale organization of brain topology, wherein even distant brain regions are connected. Indeed, the existence of these long-distance connections was shown by Hangman et al. in humans [49], as well as in other animals [50].

Mizuhara *et al.* investigated phase synchronization during a cognitive task and found that different brain regions presented phase lock behavior that lasted for at least 1 second [4, 51]. They also found relevant synchronization of both theta and beta waves, and the most relevant locked frequencies ranged between 13 and 17 Hz, which is the same frequency range where we observed SS in this study. Meanwhile, during the performance of attention-related tasks, Gross *et al.* observed phase synchronizations that fell within the same frequency range [52]. In both studies, the formation of neural networks was mediated through phase locking. The relevant difference between the Gross and Mizuhara studies and our current study is that the networks we detected appeared to be global rather than local. Furthermore, the earlier studies showed that synchronization was functionally correlated with attention and awareness, but our study focused on signals arising during sleep, which suggests that phase locking might play different roles in brain physiology.

The envelope procedure presents advantages when only a few channels are used and the distances between electrodes is large. This approach differs from the methodology presented by O’Reilly and Nielsen [33] in that reflected signal propagation in all available channels, and beyond adjacent channels, can be considered. There are also other advantages to using our procedure, primarily that it is easier to perform in clinics because fewer electrodes are needed (i.e., no dense electrode arrays) and the recommended 256 Hz sampling rate is sufficient. The velocity values presented here are also consistent with those reported in earlier studies [29, 30, 53]. The spindle dynamics has been subject of debate. Although some studies detected standard trajectories, most reports showed complex wave trajectory formation patterns that were often not reproducible. For instance, in some measurements, a wave could be seen to spiral in one moment, and in the next moment propagate in a straight line [29, 30, 33, 53]. As such, our methodology may be considered very conservative.

Previous work by Manjarrez *et al.* investigated spindle propagation by considering the signal measured at each channel as a wave packet moving at a local velocity [54]. However, even by applying the simple model proposed here, the local movement that could be observed in the channels would be complex and counter-intuitive. In general, scalp EEG wave propagation is indeed very complex since the signal first emerges at some focal point and then concomitantly spreads along the neural population. At the same time, this signal may be reaching secondary channels, which complicates the determination of the region where the signal originated.

In our case, we investigated isolated spindles with global characteristics. From results in the literature, slow SS would be expected to be more prominent in frontal channels and detected at a lower amplitude by other channels. Similarly, fast SS would likely be more prominent in parietal regions and then propagate to frontal sites. Although there are many studies that have attempted to map these propagation directions, the origin of the spindles is generally accepted to be thalamic. Therefore, we mapped the wave origin by assuming a homogeneous and isotropic system in three dimensions that allowed the verification of whether the origin of SS is congruent with physiological models.

A recent study by Frauscher *et al.* [55] evaluated SS in epileptic patients, and correlated surface measurements with intracerebral depth measures. Their results suggest that SS have a strong cortical influence and could be considered to be a local cortical event. However, those results may not be comparable to ours, since, as pointed in Nir *et al.* [46], not all SS are global and here we focused only on global SS. Our work suggests that diffuse SS can be used to characterize the thalamic source in greater detail despite the presence of many local SS. The origin of local SS can be associated with the intrinsic cortical responses such as reverberation. Considering local and global spindles together may lead to different interpretations and results when the effects of wave propagation are the focus of study.

By considering diffuse SS as global phenomena that are observed at different brain regions and yet originate in the thalamus, we may understand propagation of SS using a simple model. Excitation and suppression mechanisms also are likely to play a role in propagating SS, as evidenced by results in patients with apnea or other disorders that showed a tendency for SS to have a more constant frequency (i.e., less chirp) [27]. Recently, we investigated SS in the context of obstructive sleep apnea (OSA), and found a correlation between the degree of apnea and some quantifiers of SS such as frequency and chirp [27, 35].

Moreover, the wave propagation velocity of a field potential (which may be indirectly represented by scalp EEG) across the brain is certainly an important indirect measure of environment properties. Thus, precise measurements of the speed of waves propagating in a medium are commonly used to make inferences about the conditions of the medium itself, or about any obstacles and discontinuities that exist in the wave path. Although similar inferences can be made about EEG signals, neuron populations in the cortex oscillate and propagate this oscillation [33], which implies that neighboring neuronal populations help maintain these oscillations. One natural mechanism of synchrony can be measured by the relationship between chirp effects seen among different channels. Frequency modulation, as measured in previous studies [26, 27, 33], could be correlated to wave propagation along the cortex mass. In any case, defects in neuronal networks could easily be reflected by alterations in both chirp and propagation velocity. Therefore, separating these two effects is important for the investigation of these values as reliable pathology quantifiers.

We have only few electrodes, and using them we are directed approximately to the same place in thalamus. Studies using more electrodes would improve spatial resolution. With our resolution it is not possible to say if very close different networks are being used to generate the two different SS, but it is noteworthy that Slow SS seems to be more diffuse than Fast SS in our analysis. We believe that there are no separated global two regions, but it seems that the networks that generate both Fast and Slow spindle types lie very close.

## 5 Conclusion

In this study we found no correlation between wave velocity, synchronization amplitude and frequency modulation of global SS. In a passive medium we would expect constant wave speed, no phase locking and no frequency modulation in the neural substrate. As such, this approximation seems reasonable for investigating the envelope properties of SS, but is inappropriate for investigating the oscillatory behavior of SS, since both frequency and phase are influenced by the inherent complexity of the medium. This complexity is also reflected by the amplitude distribution of scalp SS, implying that some cortex regions are more strongly connected to the thalamic region than others, such that slow spindles and fast spindles might have different effects on different regions. The finding that slow spindles are more prone to chirp is an indication that the medium reaction indeed depends on the transmitted frequencies.

Our study supports the idea that a significant proportion of SS should be considered as a global brain phenomenon, since most characteristics of SS such as frequency, duration and chirp are strongly correlated. Moreover, there is a clear indication that the system as a whole presents high levels of synchrony. Since this collective behaviour is strongly dependent on the complex topology of the neural substrate, this method may prove to be a useful tool for characterizing disease phenotypes in neurodegenerative processes (e.g., Alzheimer’s disease) that are expected to influence synaptic strength. Thus, this method should be further developed in future studies.

## Supporting Information

S1 Data Sleep spindle characteristics data. ZIP file with all data, figures and the script used in sleep spindle analysis.
(ZIP)

## References

[1] Strogatz S. (2001) Exploring complex networks. *Nature*, 410(6825):268–276. 10.1038/35065725 11258382

[2] Crick F, Koch C (1990) Towards a neurobiological theory of consciousness. *Seminars in the Neurosciences*.

[3] Kocsis B, Prisco G V, Vertes RP (2001) Theta synchronization in the limbic system: the role of Gudden’s tegmental nuclei. *European Journal Of Neuroscience*, 13(2):381–388. 10.1111/j.1460-9568.2001.tb01708.x 11168543

[4] Mizuhara H, Yamaguchi Y (2007) Human cortical circuits for central executive function emerge by theta phase synchronization. *Neuroimage*, 36(1):232–244. 10.1016/j.neuroimage.2007.02.026 17433880

[5] Girard P, Hupé J M, Bullier J (2001) Feedforward and feedback connections between areas V1 and V2 of the monkey have similar rapid conduction velocities. *Journal of neurophysiology*, 85(3):1328–1331. 11248002 10.1152/jn.2001.85.3.1328

[6] Engel A K, Kreiter A K, König P, Singer W (1991) Synchronization of oscillatory neuronal responses between striate and extrastriate visual cortical areas of the cat. *Proceedings of the National Academy of Sciences of the United States of America*, 88(14):6048–6052. 10.1073/pnas.88.14.6048 2068083 PMC52019

[7] Rodriguez E et al (1999) Perception’s shadow: long-distance synchronization of human brain activity. *Nature*, 397(6718):430–433. 10.1038/17120 9989408

[8] Varela F, Lachaux J-P, Rodriguez E, Martinerie J (2001) The brainweb: Phase synchronization and large-scale integration. *Nature Reviews Neuroscience*, 2(4):229–239. 10.1038/35067550 11283746

[9] Buzsáki G (1996) The hippocampo-neocortical dialogue. *Cerebral Cortex*. 8670641 10.1093/cercor/6.2.81

[10] Berger H (1929) Über das elektrenkephalogramm des menschen. *Archiv für Psychiatrie und Nervenkrankheiten*, 87:527–570. 10.1007/BF01797193

[11] Subha D P, Joseph P K, Acharya U R, Lim C M (2010) EEG signal analysis: a survey. *Journal of medical systems*, 34(2):195–212. 10.1007/s10916-008-9231-z 20433058

[12] Stam C J (2005) Nonlinear dynamical analysis of EEG and MEG: review of an emerging field. *CLINICAL NEUROPHYSIOLOGY*, 116(10):2266–2301. 10.1016/j.clinph.2005.06.011 16115797

[13] Pereda E, Quiroga R Q, Bhattacharya J (2005) Nonlinear multivariate analysis of neurophysiological signals. *Progress in neurobiology*, 77(1–2):1–37. 16289760 10.1016/j.pneurobio.2005.10.003

[14] Jankel W R, Niedermeyer E (1985) Sleep Spindles. *Journal of clinical neurophysiology*, 2(1). 10.1097/00004691-198501000-00001 3932462

[15] Fogel S M, Smith C T (2011) The function of the sleep spindle: A physiological index of intelligence and a mechanism for sleep-dependent memory consolidation. *Neuroscience & Biobehavioral Reviews*, 35(5):1154–1165. 10.1016/j.neubiorev.2010.12.003 21167865

[16] Loomis A L, Harvey E N, Hobart G (1935) Potential rhythms of the cerebral cortex during sleep. *Science*. 10.1126/science.82.2122.198 17739875

[17] Inostroza M, Born J (2013) Sleep for preserving and transforming episodic memory. *Annual review of neuroscience*, 36(1):79–102. 10.1146/annurev-neuro-062012-170429 23642099

[18] Astori S, Wimmer R D, Lüthi A (2013) Manipulating sleep spindles–expanding views on sleep, memory, and disease. *Trends In Neurosciences*, 36(12):738–748. 10.1016/j.tins.2013.10.001 24210901

[19] Walker M P, Stickgold R (2014) Sleep, memory and plasticity. *Neuroscience and Psychoanalysis*.

[20] A GENERAL (2005) International Classification of Sleep Disorders, 2nd edn: *Diagnostic and Coding Manual*. Westchester, Illinois: American Academy of Sleep Medicine. Sleep.

[21] Iber C, Ancoli-Israel S, Chesson A, Quan S F (1970) *The AASM Manual for the Scoring of Sleep and Associated Events: Rules*, *Terminology and Technical Specifications*. American Academy of Sleep Medicine, American Academy of Sleep Medicine, Westchester, Illinois, 1ed edition.

[22] Schönwald S V, Carvalho D Z, Dellagustin G, de Santa-Helena E L, Gerhardt G J L (2011) Quantifying chirp in sleep spindles. *Journal of Neuroscience Methods*, 197(1):158–164. 10.1016/j.jneumeth.2011.01.025 21291911

[23] Steriade M (2000) Corticothalamic resonance, states of vigilance and mentation. *Neuroscience*, 101(2):243–276. 10.1016/S0306-4522(00)00353-5 11074149

[24] Destexhe A, Sejnowski T J (2010) Sleep oscillations. *The Handbook of Brain Theory and Neural Networks*.

[25] Destexhe A, Sejnowski, T J (2009) *Sleep and sleep states: thalamic regulation*. Encyclopedia of Neuroscience.

[26] Sitnikova E, Hramov A E, Grubov V, Koronovsky A A (2014) Age-Dependent Increase of Absence Seizures and Intrinsic Frequency Dynamics of Sleep Spindles in Rats. *Neuroscience Journal*, 2014(34):1–6. 10.1155/2014/370764 PMC4437255 26317108

[27] Carvalho D Z et al (2014) Loss of sleep spindle frequency deceleration in obstructive sleep apnea. *Clinical Neurophysiology*, 125(2):306–312. 10.1016/j.clinph.2013.07.005 23899859

[28] Ayoub A et al (2011) Differential effects on fast and slow spindle activity, and the sleep slow oscillation in humans with carbamazepine and flunarizine to antagonize voltage-dependent Na+ and Ca2+ channel activity. *Sleep*, 36(6):905–911. 10.5665/sleep.2722 PMC3649833 23729934

[29] Nunez P L (1974) The brain wave equation: a model for the {EEG}. *Mathematical Biosciences*, 21(3–4):279–297. 10.1016/0025-5564(74)90020-0

[30] Nunez P L (2000) Neocortical dynamic theory should be as simple as possible, but not simpler. *Behavioral and Brain Sciences*, 23:415–432. 10.1017/S0140525X00403250 11301576

[31] Nunez P L, Srinivasan R (2006) A theoretical basis for standing and traveling brain waves measured with human EEG with implications for an integrated consciousness. *CLINICAL NEUROPHYSIOLOGY*, 117(11):2424–2435. 10.1016/j.clinph.2006.06.754 16996303 PMC1991284

[32] Nunez P L, Srinivasan R (2014) Neocortical dynamics due to axon propagation delays in cortico-cortical fibers: EEG traveling and standing waves with implications for top-down influences on local networks and white matter disease. *Brain research*, 1542:138–166. 10.1016/j.brainres.2013.10.036 24505628 PMC3942804

[33] O’Reilly C, Nielsen T (2014) Assessing {EEG} sleep spindle propagation. part 2: Experimental characterization. *Journal of Neuroscience Methods*, 221(0):215–227. 23999173 10.1016/j.jneumeth.2013.08.014

[34] Andrillon T et al (2011) Sleep spindles in humans: insights from intracranial eeg and unit recordings. *J. Neurosci*., 31(49):17821–17834. 10.1523/JNEUROSCI.2604-11.2011 22159098 PMC3270580

[35] Schönwald S V, Carvalho D Z, de Santa-Helena E, Lemke N, Gerhardt G J L (2012) Topography-specific spindle frequency changes in obstructive sleep apnea. *BMC Neuroscience*, 13(1):89 10.1186/1471-2202-13-89 22985414 PMC3496607

[36] Durka P J, Ircha D, Blinowska K J (2001) Stochastic time-frequency dictionaries for matching pursuit. *Signal Processing*, *IEEE Transactions on*, 49(3):507–510. 10.1109/78.905866

[37] Mallat S G, Zhang Z (1993). Matching pursuits with time-frequency dictionaries. *Signal Processing*, *IEEE Transactions on*, 41(12):3397–3415. 10.1109/78.258082

[38] Mallat, S (1999) A wavelet tour of signal processing.

[39] Durka P J, Szelenberger W, Blinowska K J, Androsiuk W, Myszka M (2002) Adaptive time–frequency parametrization in pharmaco EEG. *Journal of Neuroscience Methods*, 117(1):65–71. 10.1016/S0165-0270(02)00075-4 12084565

[40] Durka P J (2003) From wavelets to adaptive approximations: time-frequency parametrization of EEG. *Biomedical engineering online*, 2:1 10.1186/1475-925X-2-1 12605721 PMC149437

[41] Schönwald S V, de Santa-Helena E L, Rossatto R, Chaves M L F, Gerhardt G J L (2006) Benchmarking matching pursuit to find sleep spindles. *Journal of Neuroscience Methods*, 156(1–2):314–321. 16546262 10.1016/j.jneumeth.2006.01.026

[42] Strogatz S H (2000) From Kuramoto to Crawford: exploring the onset of synchronization in populations of coupled oscillators. *Physica D: Nonlinear Phenomena*, 143(1–4):1–20. 10.1016/S0167-2789(00)00094-4

[43] Acebrón J, Bonilla L, Perez-Vicente C, Ritort F, Spigler R (2005) The Kuramoto model: A simple paradigm for synchronization phenomena. *Rev. Mod. Phys*., 77(1):137–185. 10.1103/RevModPhys.77.137

[44] Fox M D et al (2005) From the cover: The human brain is intrinsically organized into dynamic, anticorrelated functional networks. *Proceedings of the National Academy of Sciences*, 102(27):9673–9678. 10.1073/pnas.0504136102 PMC1157105 15976020

[45] Niedermeyer E, da Silva F H L (2005) *Electroencephalography: Basic Principles*, *Clinical Applications*, *and Related Fields*. Lippincott Williams & Wilkins.

[46] Nir Y et al (2011) Regional slow waves and spindles in human sleep. *Neuron*, 70(1):153–169. 10.1016/j.neuron.2011.02.043 21482364 PMC3108825

[47] Destexhe A, Contreras D, Sejnowski T J, Steriade M (1994) A model of spindle rhythmicity in the isolated thalamic reticular nucleus. *Journal of Neurophysiology*, 72(2):803–818. 7527077 10.1152/jn.1994.72.2.803

[48] Schabus M et al (2007) Hemodynamic cerebral correlates of sleep spindles during human non-rapid eye movement sleep. *Proceedings of the National Academy of Sciences*, 104(32):13164–13169. 10.1073/pnas.0703084104 PMC1941810 17670944

[49] Hagmann P et al (2008) Mapping the Structural Core of Human Cerebral Cortex. *PLoS Biology*, 6(7):e159–15. 10.1371/journal.pbio.0060159 18597554 PMC2443193

[50] Shanahan M, Bingman V P, Shimizu T, Wild M, Güntürkün O (2013) Large-scale network organization in the avian forebrain: a connectivity matrix and theoretical analysis. *Frontiers in computational neuroscience*, 7:89 10.3389/fncom.2013.00089 23847525 PMC3701877

[51] Mizuhara H, Wang L Q, Kobayashi K, Yamaguchi Y (2004) A long-range cortical network emerging with theta oscillation in a mental task. *Neuroreport*, 15(8):1233–1238. 15167540 10.1097/01.wnr.0000126755.09715.b3

[52] Gross J et al (2004) Modulation of long-range neural synchrony reflects temporal limitations of visual attention in humans. *Proceedings of the National Academy of Sciences of the United States of America*, 101(35):13050–13055. 10.1073/pnas.0404944101 15328408 PMC516515

[53] Ingber L, Nunez P L (2011) Neocortical dynamics at multiple scales: {EEG} standing waves, statistical mechanics, and physical analogs. *Mathematical Biosciences*, 229(2):160–173. 10.1016/j.mbs.2010.12.003 21167841

[54] Manjarrez E, Vázquez M, Flores A (2007) Computing the center of mass for traveling alpha waves in the human brain. *Brain Research*, 1145:239–247. 10.1016/j.brainres.2007.01.114 17320825

[55] Frauscher B, Ellenrieder N, Dubeau F, Gotman J (2015) Scalp spindles are associated with widespread intracranial activity with unexpectedly low synchrony. *NeuroImage*, 105:1–12. 10.1016/j.neuroimage.2014.10.048 25450108 PMC4275575

