# Supplementary figures and images for: Synchronization and Propagation of Global Sleep Spindles
Source: PLoS One. 2016 Mar 10;11(3):e0151369. doi: 10.1371/journal.pone.0151369 (PMC4786112; doi:10.1371/journal.pone.0151369)

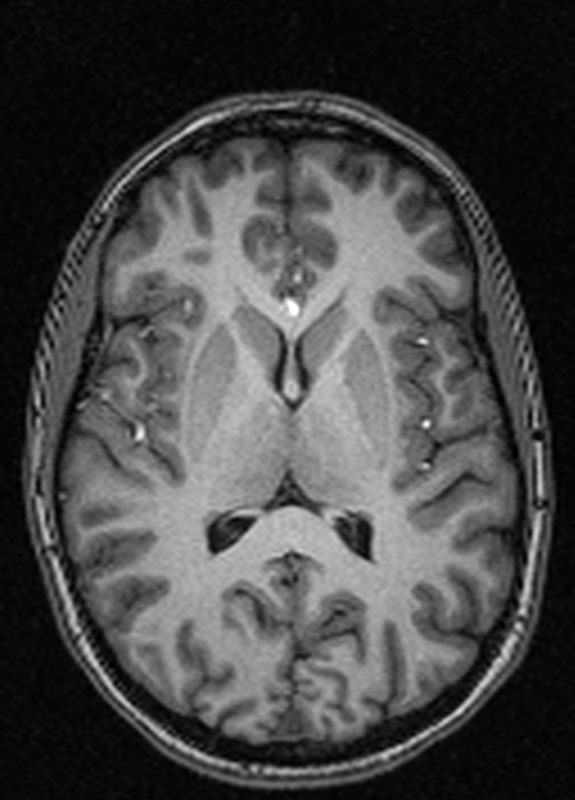

Supplement: S1 Data — ZIP file with all data, figures and the script used in sleep spindle analysis. (ZIP) [file pone.0151369.s001.zip › figures/N_Axial_Fig.png]

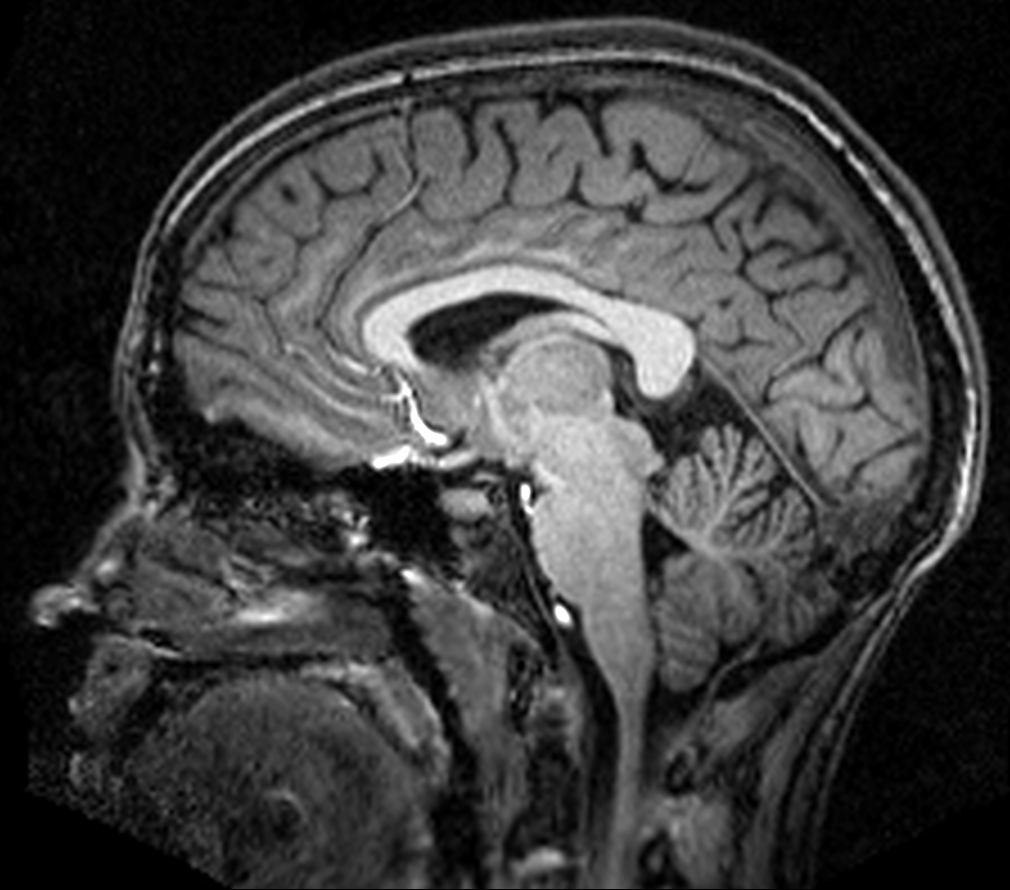

Supplement: S1 Data — ZIP file with all data, figures and the script used in sleep spindle analysis. (ZIP) [file pone.0151369.s001.zip › figures/N_Sagit_Fig.png]
